# Mouse prefrontal cortex represents learned rules for categorization

**DOI:** 10.1038/s41586-021-03452-z

**Published:** 2021-04-21

**Authors:** Sandra Reinert, Mark Hübener, Tobias Bonhoeffer, Pieter M. Goltstein

**Affiliations:** 1 Max Planck Institute of Neurobiology, Martinsried, Germany; 2 Graduate School of Systemic Neurosciences, Ludwig-Maximilians-Universität München, Martinsried, Germany

**Keywords:** Learning and memory, Cortex, Long-term memory, Operant learning, Neural circuits

## Abstract

The ability to categorize sensory stimuli is crucial for an animal’s survival in a complex environment. Memorizing categories instead of individual exemplars enables greater behavioural flexibility and is computationally advantageous. Neurons that show category selectivity have been found in several areas of the mammalian neocortex^1–4^, but the prefrontal cortex seems to have a prominent role^4,5^ in this context. Specifically, in primates that are extensively trained on a categorization task, neurons in the prefrontal cortex rapidly and flexibly represent learned categories^6,7^. However, how these representations first emerge in naive animals remains unexplored, leaving it unclear whether flexible representations are gradually built up as part of semantic memory or assigned more or less instantly during task execution^8,9^. Here we investigate the formation of a neuronal category representation throughout the entire learning process by repeatedly imaging individual cells in the mouse medial prefrontal cortex. We show that mice readily learn rule-based categorization and generalize to novel stimuli. Over the course of learning, neurons in the prefrontal cortex display distinct dynamics in acquiring category selectivity and are differentially engaged during a later switch in rules. A subset of neurons selectively and uniquely respond to categories and reflect generalization behaviour. Thus, a category representation in the mouse prefrontal cortex is gradually acquired during learning rather than recruited ad hoc. This gradual process suggests that neurons in the medial prefrontal cortex are part of a specific semantic memory for visual categories.

## Abstract (web summary)

Neurons in the mouse medial prefrontal cortex acquire category-selective responses with learning.

## Main

**Fig. 1:**
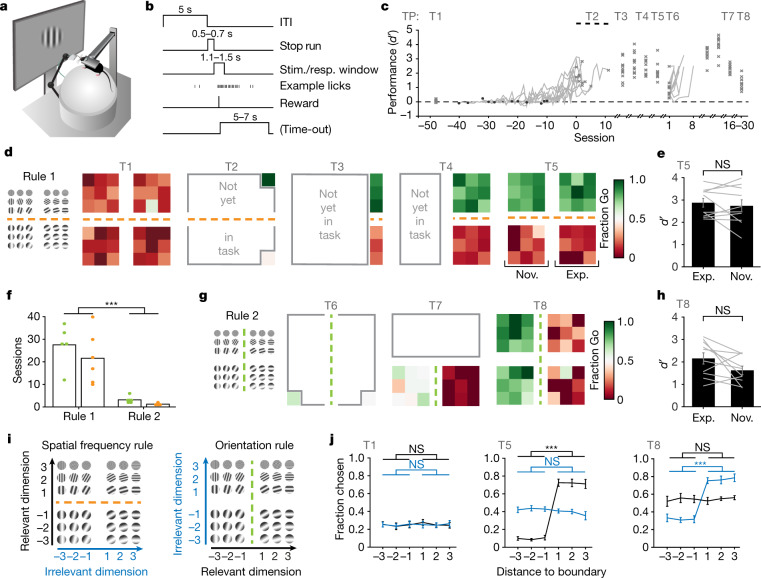
Mice learn rules to categorize visual stimuli and generalize to novel stimuli. **a**, Schematic of behavioural training setup. **b**, Schematic of trial structure in the Go/NoGo task. ITI, inter-trial interval; Stim./resp., stimulus presentation/response window. **c**, Performance (*d*′) of 11 mice in each training session. Individual traces aligned to criterion (66% of correct trials). The dashed line indicates chance level (*d*′ = 0). Crosses denote sessions with two-photon imaging (T1–T8). The spread in performance after T2 is due to day-to-day variability rather than mouse-to-mouse variability. TP, time point. **d**, Fraction of Go choices per stimulus of an example mouse at each time point (of two-photon imaging) until the presentation of all 36 stimuli of rule 1 (generalization; T5). **e**, Performance (*d*′) for rule 1 (T5), for experienced (Exp.) compared to novel (Nov.) stimuli. *P* = 0.50, two-tailed paired-samples *t*-test (*n* = 11 mice). Grey lines denote individual mice. Data are mean ± s.e.m. **f**, Number of training sessions until criterion (66% correct, exemplar stimuli). Bars indicate mean across mice, dots are individual mice (green denotes the orientation rule; orange denotes spatial frequency rule). Rule 2 is learned significantly faster than rule 1. *P* = 9.77 × 10^−4^, two-tailed Wilcoxon matched-pairs signed-rank (WMPSR) test (*n* = 11 mice). **g**, As in **d**, for rule 2 of the same mouse. **h**, As in **e** for rule 2 (T8). *d*′ did not differ significantly between novel stimuli and stimuli experienced with rule 2. *P* = 0.09, two-tailed paired-samples *t*-test (*n* = 10 mice). **i**, Schematics specifying the distance of stimuli to the boundary. **j**, Psychometric curves showing the fraction of Go choices along the relevant (black) and irrelevant (blue) dimension of rule 1 at T1, T5 and T8. Left: *P*_relevant T1_ = 0.36, *P*_irrelevant T1_ = 0.77; middle: ***P_relevant T5_ = 1.73 × 10^−6^, *P*_irrelevant T5_ = 0.09; right: *P*_relevant T5_ = 0.73, ***P_irrelevant T5 (relevant T8)_ = 1.73 × 10^−6^, two-tailed WMPSR test, Bonferroni-corrected for two comparisons (*n* = 10 mice). Categorization performance was not affected by the order in which mice were trained on orientation and spatial frequency rules. Data are mean ± s.e.m. across mice; for individual mice, see Extended Data Fig. 2. NS, not significant. Source data

**Extended Data Fig. 1:**
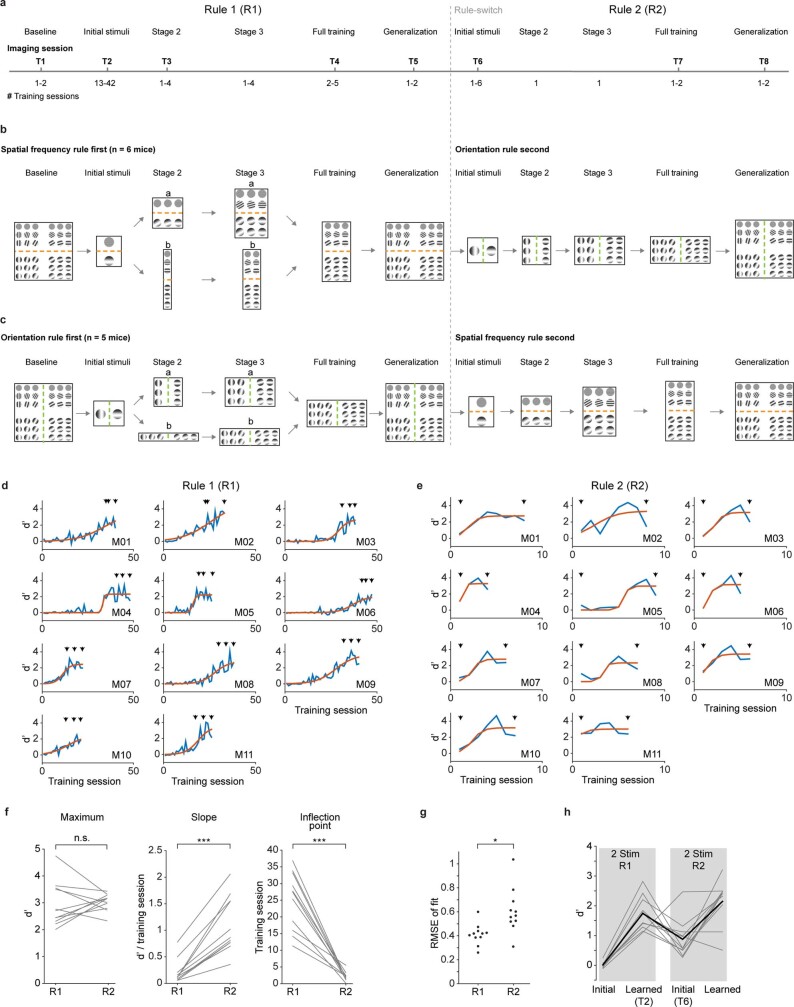
Timeline of behavioural training, presented stimuli and learning performance of individual mice for both categorization rules. **a**, Timeline showing behavioural training stages, the number of training sessions that mice spent in each stage (min–max) and the imaging sessions (T1–T8). **b**, Stimuli used for category training, aligned to the stages shown in **a**. The scheme shows stimuli for mice that were trained on the spatial frequency rule first, and the orientation rule second. **c**, As in **b**, but for mice trained on the orientation rule first. **d**, Per mouse, the learning curve for training on rule 1. Blue curve denotes single session *d*′. Orange curve denotes sigmoid fit of *d*′. Arrows indicate imaging time points T2, T3 and T4. **e**, As in **d**, but for rule 2. Arrows indicate imaging time points T6 and T7. **f**, Parameters describing the fitted sigmoidal curves, comparing rules 1 and 2. Left, maximum. *P* = 0.71, two-tailed paired-samples *t*-test (*n* = 11). Middle, slope. *P* = 1.9 × 10^−5^, two-tailed paired-samples *t*-test (*n* = 11). Right, inflection point. *P* = 9.3 × 10^−6^, two-tailed paired-samples *t*-test (*n* = 11). **g**, Root-mean-square error (RMSE) of sigmoid fit. *P* = 0.013, two-tailed paired-samples *t*-test (*n* = 11). **h**, *d*′ of all mice comparing naive and learned discrimination of the initial two stimuli for the first rule (left) and the second rule (right). Black line indicates the mean across all mice, grey lines represent data of individual mice. Source data

**Extended Data Fig. 2:**
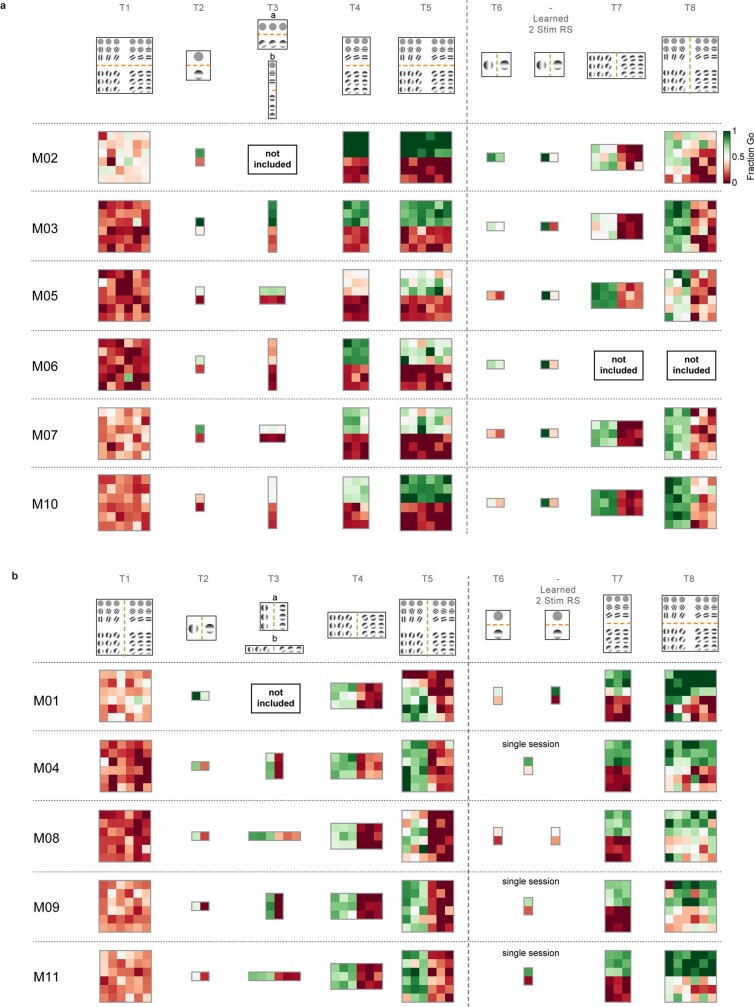
Categorization, generalization and rule-switch performance for individual mice. Performance as the fraction of Go choices per stimulus, averaged over the imaging time points for each mouse individually. The time point ‘Learned 2 stim RS’ shows performance after the rule-switch was successfully learned. This time point was not an imaging session. Three mice learned the rule-switch during session T6 (‘single session’). **a**, Mice first trained on the spatial frequency rule and then on the orientation rule (data of M03 is also shown in Fig. 1d, g). **b**, As in **a** for all mice trained initially on the orientation rule and then on the spatial frequency rule. Mouse M06: imaging sessions T7 and T8 were ‘not included’ owing to poor imaging quality; Mice M01 and M02: imaging session T3 was not recorded. Source data

We trained head-fixed mice (*n* = 11) in a ‘Go’/‘NoGo’ rule-based categorization task (Fig. 1a, b) to sort visual stimuli into two groups. Stimuli were 36 sinusoidal gratings, each with a specific combination of two stimulus features: orientation and spatial frequency. At any time, one rule determined the relevant feature for categorization (that is, the active rule; for example, assigning category identity of a stimulus based on orientation)^10,11^ (Extended Data Fig. 1). First, mice learned to discriminate two exemplar stimuli according to the active rule. All mice achieved more than 66% correct Go choices within 10 to 40 sessions, showing considerable variability in the rate of learning. We then introduced categories by stepwise addition of stimuli to the task, up to a set of 18 different gratings that varied along both feature dimensions, orientation and spatial frequency (Extended Data Fig. 1b, c). Mice integrated the newly introduced stimuli within 1 to 2 sessions and they maintained a sensitivity index *d*′ of >1 (Fig. 1c, d, Extended Data Figs. 1d, 2).

## Mice learn to categorize visual stimuli

**Extended Data Fig. 3:**
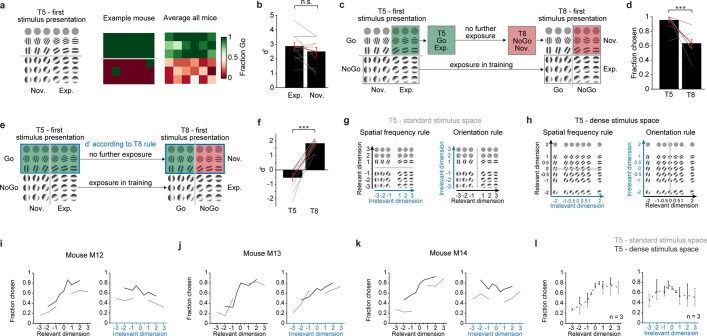
Generalization of stimuli at their first presentation and categorization of stimuli close to, and at the category boundary. **a**, Left, schematic of stimulus space during the generalization session (T5). Middle, category choice for every stimulus on its first presentation for an example mouse, green: Go choice, red: NoGo choice. Right, category choice at the first stimulus presentation, averaged across mice (*n* = 10 mice). **b**, *d*′ for experienced stimuli and novel stimuli separately, calculated using only the first presentation of each stimulus at the generalization session T5. *P* = 0.19, two-tailed paired-samples *t*-test (*n* = 10). Grey lines denote individual mice. Data are mean ± s.e.m. (across mice). **c**, Mice use the second rule to categorize stimuli that were only experienced during training on the first rule. Left, schematic showing category identity of stimuli at T5 (Go or NoGo) and whether they were experienced throughout category training on rule 1 (Exp) or novel (Nov). Middle, the highlighted quadrant (green) was part of the Go category; stimuli from this quadrant had been incrementally used throughout category learning up to T5. After T5, mice were trained on the second rule, using only stimuli in the bottom half of the category space (which corresponded to the NoGo category at T5). Right, in the second generalization session (T8, rule-switch generalization), mice were once more exposed to the full category space. Now, the same highlighted quadrant (red) required a NoGo response. However, so far these stimuli were extensively (and only) experienced as requiring a Go response. If mice showed a different fraction of Go choices in T5 and T8, it would reflect category generalization of rule 2, because the absence of experience with the stimuli in the highlighted quadrant prevented learning of a stimulus-response mapping. **d**, Fraction of Go category choices for the first presentation of stimuli highlighted in **c**, comparing T5 and T8. Data are mean ± s.e.m. (fraction chosen across all mice). Grey lines indicate data of individual mice, of which some overlap. *P* = 0.002, two-tailed paired-samples *t*-test (*n* = 10). **e**, Schematic as in **c**, indicating the stimuli and the rule for which *d*′ was calculated in **f**. **f**, *d*′ for the first presentation of stimuli highlighted in **c**, comparing T5 and T8. Grey lines denote individual mice. *P* = 2.2 × 10^−5^, two-tailed paired-samples *t*-test (*n* = 10). Data are mean ± s.e.m. (across mice). **g**, Schematic indicating the relevant and irrelevant stimulus dimension for the spatial frequency rule (left) and the orientation rule (right) at T5. **h**, As in **g**, for training with a dense stimulus space (*n* = 3 mice) to determine categorization behaviour closer to the category boundary. **i**–**k**, Psychometric curves for the three individual mice. The fraction chosen (fraction of Go choices) is shown along the relevant dimension (left) and the irrelevant dimension (right). Grey lines denote data from the T5 generalization session. Black lines denote data from the session with the dense stimulus space. **l**, As in **i**, showing the mean (± s.e.m.) across the three mice (shown in **i**, **j** and **k**) that were tested using the dense stimulus space. Source data

To determine whether mice had indeed learned categorization, we tested a characteristic feature of category learning, rapid generalization to novel stimuli^10–13^. Mice were presented with 18 novel grating stimuli in addition to the 18 experienced ones. All mice were able to generalize the learned rule to novel stimuli upon their first presentation (time point 5, T5) (Fig. 1d, Extended Data Fig. 3a), performing equally well on novel and experienced stimuli (Fig. 1e, Extended Data Fig. 3b).

Because rule-switching is key to rule-based categorization^11,14,15^, our stimulus set was designed to allow for testing this aspect. Thus, after learning the first rule, mice were required to group the same stimuli into new categories according to a new rule, by making the previously irrelevant stimulus feature (for example, spatial frequency) relevant and the relevant one (for example, orientation) irrelevant. All mice learned to discriminate two exemplar stimuli for rule 2 considerably faster than during initial learning (Fig. 1f, Extended Data Fig. 1e–h). After the mice had learned to categorize a set of 18 stimuli according to rule 2, they were able to generalize to the 18 stimuli they had so far experienced only with rule 1 (Fig. 1g, h, Extended Data Fig. 3c–f). We tested whether there were any residual effects of the former rule on the categorization behaviour of the mice by comparing the influence of each stimulus feature (Fig. 1i) on the choices of the mice before learning (time point T1) and after learning each rule (T5 and T8). Untrained mice showed no effect of either stimulus feature on categorization (Fig. 1j, left). Trained mice only based categorization on the stimulus feature relevant to the active rule; the irrelevant feature showed no effect (Fig. 1j, middle, right; for a detailed analysis of categorization near the boundary see Extended Data Fig. 3g–l). In summary, all mice learned discriminating categories on the basis of two different rules, and they generalized these rules to novel stimuli, probably by selectively attending^16^ to the relevant stimulus feature. Having established this training paradigm, we began tracking neuronal correlates of rule-based categories throughout learning.

## mPFC contains category-selective neurons

**Fig. 2:**
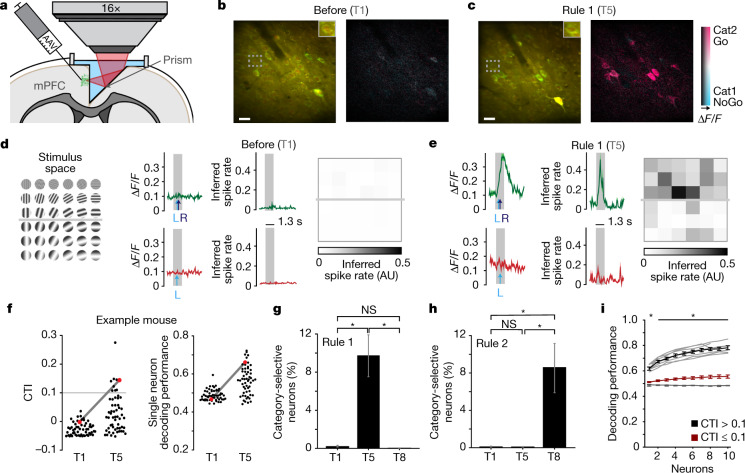
Single neurons in the mouse mPFC develop category-selective responses. **a**, Schematic depicting virus injection into the mPFC and two-photon imaging through a prism implant between the hemispheres, adapted from ref. ^29^. AAV, adeno-associated virus. **b**, Example field of view before learning (T1). Left, pseudo-coloured GCaMP6m (green) and mRuby2 (red) fluorescence. Right, hue, saturation and lightness (HLS) map. Hue: preferred category; lightness: response amplitude; saturation: category selectivity. Scale bar, 30 μm. **c**, As in **b**, after learning (T5). **d**, Left, stimulus space. Middle, average response to stimuli of the cell highlighted in **b** (Δ*F*/*F*, green: Go category, red: NoGo category). L, average time of first lick. R, average time of reward delivery. Right, inferred spike rate. AU, arbitrary units. **e**, As in **d**, after learning, showing selective responsiveness to Go category stimuli. **f**, Left, CTI of all cells in the example field of view before and after learning. Red, example cell in **b**. Grey line denotes the threshold used for further analyses (CTI > 0.1). Right, cross-validated performance of a Bayesian decoder predicting the category of the presented stimulus. CTI correlates with decoding performance. *P* = 2.2 × 10^−10^, rho = 0.41, Spearman’s correlation (*n* = 213 category-selective neurons, CTI > 0.1). **g**, Percentage of category-selective cells for rule 1. T1, before learning, rule 1 active. T5, after learning, rule 1. T8, after learning, rule 2. *P*_T1–T5_ = 0.006, *P*_T1–T8_ = 0.25, *P*_T5–T8_ = 0.004, two-tailed WMPSR test, Bonferroni-corrected for three comparisons (*n*_mice_ = 10, *n*_neurons_ = 2,306). **h**, As in **g**, for rule 2. *P*_T1–T5_ = 0.75, *P*_T1–T8_ = 0.004, *P*_T5–T8_ = 0.004, two-tailed WMPSR test, Bonferroni-corrected for three comparisons (*n* = 10 mice). **i**, Bayesian decoding performance as in **f**, for all mice. Data are shown separately for populations of low (red) and high (black) CTI cells. Light grey denotes individual mice; dark grey denotes average performance after shuffling stimulus categories. *P*_1 neuron_ = 0.005, *P*_2–8 neurons_ = 0.01, two-tailed WMPSR test, high versus low CTI cells, Bonferroni-corrected for two comparisons (*n*_1 neuron_ = 10 mice, *n*_2–8 neurons_ = 8 mice). Data are mean ± s.e.m. across mice; for individual mice see Extended Data Figs. 5, 6. Source data

**Extended Data Fig. 4:**
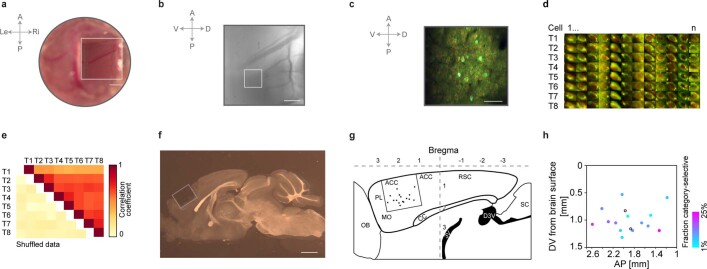
Reconstruction of the location of imaging regions. **a**, Top down view onto the craniotomy of M07 with prism implant (white square denotes the prism outline). A, anterior; P, posterior; Le, left; Ri, right. Scale bar, 0.5 mm. **b**, View through the prism with the position of an imaging field (white box), D, dorsal; V, ventral. Scale bar, 0.3 mm **c**, The imaging field in **b**, visualized with a two-photon microscope (red: structural marker mRuby2; green: functional marker GCaMP6m; image is the average of all frames of session T1). Scale bar, 30 μm. **d**, Cropped images showing 12 example neurons across all imaging time points (T1–T8). **e**, The top triangle shows the correlation between cropped images of any two time points (average across all neurons). The bottom triangle shows the correlation after shuffling cell identities (control). **f**, Example sagittal brain section showing the position of the prism implant along the anterior-posterior axis. Scale bar, 1 mm. **g**, Schematic of cortical midline regions near the prism implant (ML 0.12), modified from Franklin & Paxinos^64^, figure 102, with permission from Academic Press (Copyright 2007). 3V, third ventricle; ACC, anterior cingulate cortex; CC, corpus callosum; D3V, dorsal third ventricle; MO, medial orbital cortex; OB, olfactory bulb; PL, prelimbic cortex; RSC, retrosplenial cortex; SC, superior colliculus. The centres of all imaging regions included in Fig. 2g, h are indicated by black dots. **h**, Fraction of category-selective cells for each imaged field of view (included in Fig. 2g, h). The black, hollow circles are imaging regions without category-selective cells. There was no clear relationship between the location of the imaging regions within mPFC and the fraction of category-selective cells. Source data

**Extended Data Fig. 5:**
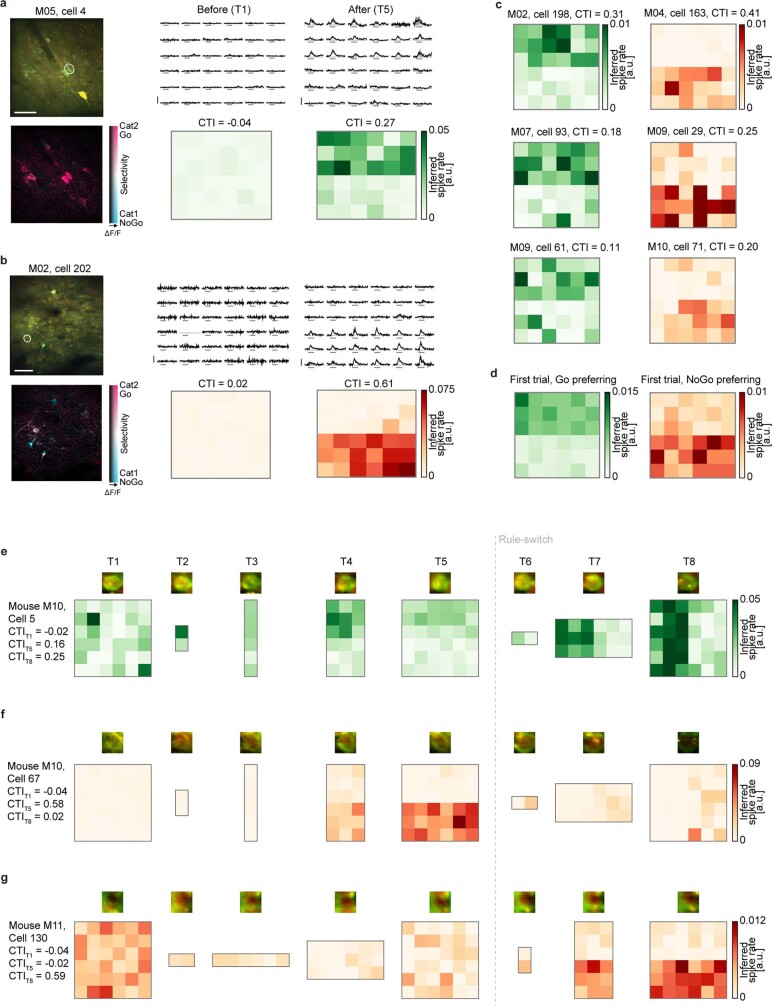
Examples of single cells that became category-selective over the course of learning. **a**, Example Go category-selective cell (from the mouse shown in Fig. 2). Top left, position of the cell in the two-photon image. Scale bar, 30 μm. Bottom left, HLS map of the example region (hue: category identity of the presented stimuli; cyan, category 1; pink, category 2; lightness: response amplitude; saturation: selectivity for the stimulus category). Top middle, before learning (T1), Δ*F*/*F* traces aligned to stimulus onset. Scale bar, 100% Δ*F*/*F*. Grey bar denotes stimulus presentation, 1.3 s. Bottom middle, mean inferred spike rate per stimulus. Right, after learning (T5). **b**, As in **a** for an example NoGo category-selective cell. **c**, Mean inferred spike rate per stimulus for six further category-selective cells from different mice. **d**, Response amplitude during the first presentation of each stimulus, averaged across all category-selective cells at T5. Left, green: Go-preferring neurons; right, red: NoGo-preferring neurons. **e**, Top row, 40 by 40 pixel cropped images showing a Go category-selective cell in the averaged two-photon imaged field of views (pseudo-coloured). Bottom row, mean inferred spike rate of the response of the example cell to the presented stimuli at each imaging time point. **f**, **g**, As in **e**, but for different, NoGo category-selective cells. Source data

The prefrontal cortex (PFC) in primates and rodents is important for cognitive functions such as categorization^6,7,16,17^, rule learning^1,18,19^ and cognitive flexibility^20–22^, even though the functional and anatomical analogy of this region across species is still debated^23–28^. Earlier studies in primates have described individual PFC neurons coding for visual categories^3,6,7^. Encouraged by this finding, we tested whether the mouse medial PFC (mPFC) contained neurons that reflected the ability of the mouse to categorize visual stimuli as described above. To this end, we chronically monitored neuronal activity in cortical layer 2/3 using two-photon calcium imaging through a microprism implant inserted between the two hemispheres, which enabled optical access to mPFC^29^ (Fig. 2a–c, Extended Data Fig. 4). We measured neuronal activity of individual cells while the mice performed the task (*d*′ ranging from 0.7 to 3.6; for imaging time points and precise trial structure, see Fig. 1b, c, Extended Data Fig. 1a). In naive mice (time point T1), mPFC neurons did not respond to visual stimuli (Fig. 2b, d, Extended Data Fig. 5), but some of these initially non-selective cells clearly showed category selectivity after learning (T5, rule 1) (Fig. 2c, e, Extended Data Fig. 5; neural correlates of other task-related aspects are described below).

**Extended Data Fig. 6:**
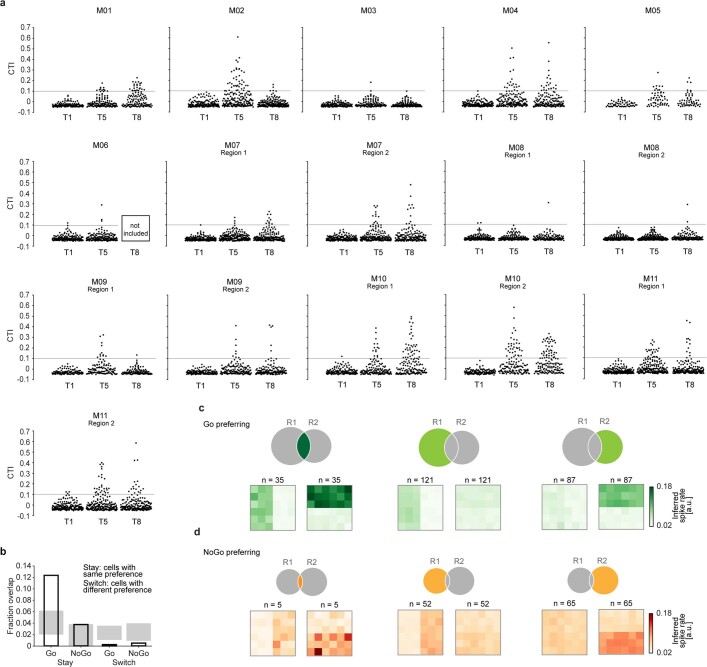
Category-tuning index distributions of all recorded field of views at T1, T5 and T8 and the overlap of populations of category-selective cells. **a**, Category-tuning index before learning (T1, according to rule 1), after the mouse had learned rule 1 (T5, according to rule 1) and after it had learned to categorize stimuli according to rule 2 (T8, according to rule 2). Each imaging region is displayed individually. Individual cells are represented as dots. Only cells recorded at all imaging time points were included. Grey line indicates the threshold CTI value of 0.1 that was applied to classify cells as category-selective. **b**, Black, the fraction of overlap between category-selective groups found at T5 and T8 (Go stay/NoGo stay: Go/NoGo category-selective at both T5 and T8; Go switch: Go category-selective at T5 and NoGo category-selective at T8; NoGo switch: NoGo category-selective at T5 and Go category-selective at T8). Grey denotes 95% confidence intervals of chance population overlap (Methods). **c**, Go category-selective cells. Top row, Venn diagrams of the fraction of cells that were category-tuned only for rule 1 (R1), only for rule 2 (R2) or for both rules (area between R1 and R2). The highlighted part of the Venn diagram indicates which data are shown in the bottom row. Bottom row, mean stimulus response amplitude (inferred spike rate) after rule 1 (left) or rule 2 (right) was learned. **d**, As in **c**, for NoGo category-selective cells. Source data

We quantified the category selectivity of individual cells using the category-tuning index (CTI)^30^, with values close to 1 indicating strong differences in activity between, but not within, categories, and a value of 0 indicating no difference in the firing rate between and within categories. We defined neurons with a CTI value above 0.1 as category-selective (Methods), and verified that these cells reliably encoded categories using cross-validated Bayesian decoding (Fig. 2f, i). In naive mice, hardly any cells exceeded this threshold, whereas after learning, a substantial fraction of neurons in the mPFC showed category selectivity (before: 0.03% ± 0.03%, after: 9.8% ± 2.2% (mean ± s.e.m.)) (Fig. 2g, Extended Data Fig. 6a).

After having learned the rule-switch, a similar fraction of cells showed selectivity for the new categories, whereas selectivity for the old, now irrelevant categories ceased (rule 1: 0.07% ± 0.05%, rule 2: 8.6% ± 2.8%) (Fig. 2h, Extended Data Fig. 6a).

To convert an internal category representation into a motor decision, it would be sufficient for cells in the mPFC to show selectivity for only one category^31^. However, we observed two types of neuron—one that represented rewarded stimuli (Go preferring: 73% of all category-selective cells at T5 and 65% at T8) and the other non-rewarded stimuli (NoGo preferring: 27% at T5 and 35% at T8). Thus, cells in the mouse mPFC develop flexible representations of rule-based categories over the course of learning.

## Category selectivity emerges over time

**Fig. 3:**
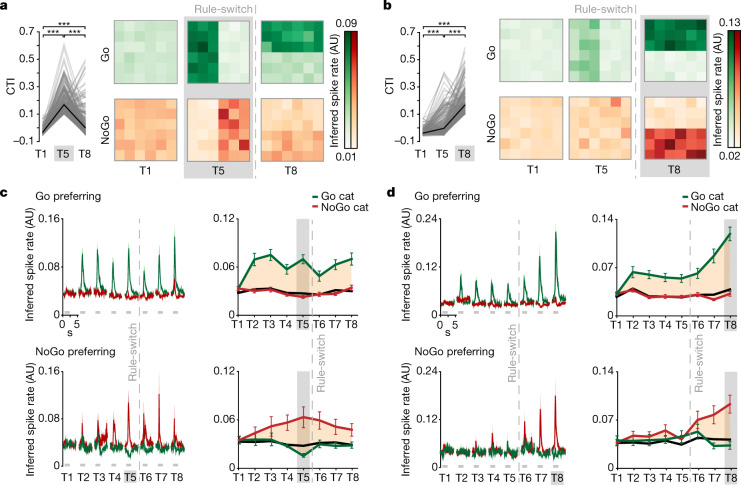
Two populations of category-selective neurons show different dynamics during a rule-switch. **a**, Left, CTI of all category-selective neurons identified at T5 (grey highlight), shown for time points T1, T5 and T8. *P*_T1–T5_ = 1.1 × 10^−36^, *P*_T1–T8_ = 1.6 × 10^−14^, *P*_T5–T8_ = 6.9 × 10^−27^, two-tailed WMPSR test, Bonferroni-corrected for three comparisons (*n* = 213 cells). Black line denotes the mean. Right, average inferred spike rate per stimulus of Go and NoGo category-selective cells at T1, T5 and T8 (*n*_Go_ = 156 cells; *n*_NoGo_ = 57 cells) **b**, As in **a**, but for category-selective cells defined at T8. *P*_T1–T5_ = 4.2 × 10^−18^, *P*_T1–T8_ = 2.9 × 10^−33^, *P*_T5–T8_ = 1.1 × 10^−27^, two-tailed WMPSR test, Bonferroni-corrected for three comparisons (*n* = 192 cells; *n*_Go_ = 122 cells; *n*_NoGo_ = 70 cells). **c**, Left, inferred spike rate of all Go (top) and NoGo (bottom) category-selective cells, identified at T5 (grey highlight), during trials of all Go (green) and NoGo (red) category stimuli at T1–T8. Grey denotes stimulus presentation. Data are mean ± s.e.m., across cells. Right, inferred spike rate during stimulus presentation of all Go (top) and NoGo (bottom) category-selective cells. Green denotes Go category, red denotes NoGo category, orange area denotes the difference. Black denotes the mean inferred spike rate in the pre-stimulus period. Data are mean ± s.e.m., across cells. **d**, As in **c**, for category-selective cells defined at T8. Source data

**Extended Data Fig. 7:**
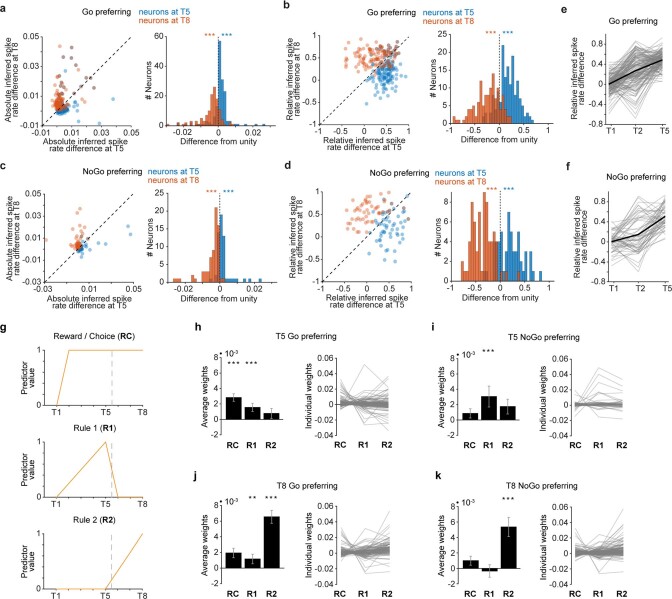
Individual neurons follow characteristic time courses of acquiring selectivity. **a**, Left, scatter plot showing the difference in mean inferred spike rate between stimuli of the two categories, after learning the first rule (T5, *x* axis) and the rule-switch (T8, *y* axis) for individual Go category-selective cells at session T5 (blue) and T8 (orange). Right, histogram of the differences from unity of the distributions shown on the left. *P*_T5_ = 1.5 × 10^−8^, *P*_T8_ = 3.5 × 10^−15^, two-tailed WMPSR (*n*_T8_ = 122, *n*_T5_ = 156). **b**, As in **a**, but showing the relative spike rate difference (normalized by the sum of inferred spike rate to category 1 and 2 stimuli) for individual Go category-selective neurons at T5 and T8. *P*_T5_ = 4.9 × 10^−21^, *P*_T8_ = 1.5 × 10^−18^, two-tailed paired-samples *t*-test (*n*_T5_ = 156, *n*_T8_ = 122). **c**, As in **a**, but for NoGo category-selective cells at T5 and T8. *P*_T5_ = 9.1 × 10^−5^, *P*_T8_ = 1.0 × 10^−12^, two-tailed WMPSR (*n*_T5_ = 57, *n*_T8_ = 70). **d**, As in **b**, but for NoGo category-selective cells. *P*_T5_ = 9.6 × 10^−8^, *P*_T8_ = 1.7 × 10^−21^, two-tailed paired-samples *t*-test (*n*_T5_ = 57, *n*_T8_ = 70). **e**, Development of the spike rate difference up to T5, for individual Go category-selective neurons at T5. Before learning, baseline: T1. After learning the initial stimuli: T2. After learning categorization: T5. Grey lines denote individual neurons. Black line denotes the mean across cells. **f**, As in **e**, but for NoGo category-selective neurons. **g**, Schematic showing predicted time courses for the acquisition of reward/choice (RC) selectivity, and category selectivity according to each rule (R1, R2). These predictors were fit to the time courses of individual neurons using linear regression in **h**–**k**. **h**, Left, mean (± s.e.m.) predictor weight of T5 Go category-selective neurons. *P*_RC_ = 3.2 × 10^−9^, *P*_R1_ = 2.0 × 10^−7^, *P*_R2_ = 0.12, two-tailed WMPSR tests, Bonferroni-corrected for three comparisons (*n* = 156). Right, the predictor weights of individual neurons. Selectivity of Go-preferring neurons was best predicted by reward/choice, and also showed a category component. **i**, As in **h**, for T5 NoGo category-selective cells. *P*_RC_ = 0.03, *P*_R1_ = 0.001, *P*_R2_ = 0.03, two-tailed WMPSR tests, Bonferroni-corrected for three comparisons (*n* = 57). Selectivity of NoGo-preferring neurons corresponded best to the time course of acquiring category rule 1. **j**, As in **h**, for Go category-selective cells defined at T8 *P*_RC_ = 0.03, *P*_R1_ = 0.003, *P*_R2_ = 7.8 × 10^−15^, two-tailed WMPSR tests, Bonferroni-corrected for three comparisons (*n* = 122). **k**, As in **h**, for NoGo category-selective cells defined at T8 *P*_RC_ = 0.09, *P*_R1_ = 0.52, *P*_R2_ = 8.0 × 10^−7^, two-tailed WMPSR tests, Bonferroni-corrected for three comparisons (*n* = 70). The best predictor for both Go and NoGo preferring category-selective neurons after the rule-switch was the gradual acquisition of category rule 2. Source data

Our chronic recording approach allowed us to ask whether the cells that coded for learned categories in rule 2 were the same ones that had represented categories in rule 1. Although many cells that were category-selective for rule 1 were less selective for rule 2, a subset of neurons remained category-selective throughout (Fig. 3a, Extended Data Fig. 7a, b). We found that, on average, the Go category-selective neurons remapped their responses to the new Go category—that is, after the rule-switch, they responded to a different set of visual stimuli. By contrast, the NoGo category-selective cells did not remap (Fig. 3a, Extended Data Fig. 7c, d). They lost their selectivity after the rule-switch, and a new set of cells became NoGo category-selective for the newly defined categories. Similarly, the Go category-selective cells observed after the rule-switch showed previous selectivity to the first rule, whereas rule 2 NoGo category-selective neurons did not show any selectivity before the rule-switch on average (Fig. 3b). In line with this, we observed that the Go category-selective populations for each rule overlapped more than expected by chance (Methods), in contrast to NoGo category-selective populations (Extended Data Fig. 6b–d). Notably, neurons were less likely than chance to switch their preference from Go to NoGo and vice versa (Extended Data Fig. 6b).

It is currently debated whether such flexible representations in the PFC are gradually built up during learning—that is, are part of the memory of the learned categories—or whether they are instantaneously assigned during the task to represent anything that becomes relevant to the animal^7–9^. This question can be answered only by monitoring neurons throughout the learning process, starting from a naive animal. We took advantage of the fact that our mice had never been trained on a categorization task and we followed the development of category-selective responses of individual neurons over the entire time course of rule-based category learning (Fig. 3c). Focusing on the period over which selectivity emerged, we observed a marked difference between the time courses that the Go and NoGo category-selective neurons followed. On average, the Go category-selective cells showed large, stable responses for the Go category, early on after presentation of the initial category stimuli in an ad hoc fashion (T2–T5) (Fig. 3c, Extended Data Fig. 7e). By contrast, the NoGo category-selective cells only gradually developed selectivity with increasing categorization demand of the task (T4, T5) (Fig. 3c, Extended Data Fig. 7f). After the rule-switch, the Go category-selective cells on average switched their stimulus selectivity, thereby retaining category selectivity. Former NoGo category-selective cells gradually lost selectivity, whereas a new, independent population of NoGo category-selective neurons gained selectivity (Fig. 3c, d). Notably, after the rule-switch, Go category-selective neurons showed increased Go responsiveness beyond a stable level of Go selectivity during earlier training (Fig. 3d).

**Extended Data Fig. 8:**
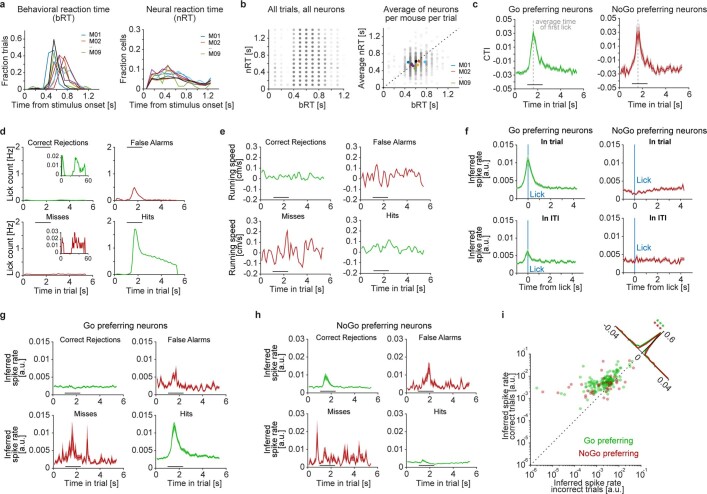
Relation between motor behaviour and neuronal responses of category-selective cells. **a**, Line histograms showing the count probability of behavioural (left) and neural (right) reaction times of individual mice. Behavioural reaction time (bRT) was measured as the time of the first lick after stimulus onset, neural reaction time (nRT) as the time of the neuronal response onset after stimulus onset. **b**, Left, scatter plot of bRT and nRT for every trial of every mouse in session T5. *P* = 2.3 × 10^−13^, rho = 0.08, Spearman’s correlation (*n* = 9,348 measured reaction times). Right, grey circles: scatter plot showing the average nRT (that is, the nRT averaged across all Go category-selective neurons, but separated per mouse and trial) versus the bRT per mouse and trial. *P* = 6.2 × 10^−6^, Pearson’s *r* = 0.13 (*n* = 1,156 trials). The density of grey circles is indicated by the colour intensity (alpha value). Coloured circles: the overall mean nRT and bRT of each mouse. *P* = 0.51, Pearson’s *r* = 0.26 (*n* = 9 mice). Dashed line denotes the unity line. **c**, CTI of Go (left) and NoGo (right) category-selective neurons, calculated for every imaging frame individually. Data show the period from 1 s before stimulus onset to 3 s after stimulus offset. Grey dashed line denotes the average time of first lick. Black line denotes the average period of stimulus presentation. **d**, Mean lick frequency in session T5, grouped by trial outcome (hits, misses, correct rejections and false alarms). Insets show the same data with inflated *y* axis. Black line denotes the average period of stimulus presentation. **e**, As in **d**, but showing the average running speed. **f**, Inferred spike rate of Go (left) and NoGo (right) category-selective neurons aligned to the onset of lick-bouts. Top row, lick-bouts detected within a trial. Bottom row, lick-bouts detected in the inter-trial-interval. Data are mean ± s.e.m. **g**, Inferred spike rate of Go category-selective neurons in session T5, grouped by trial outcome (hits, misses, correct rejections and false alarms). Black line denotes stimulus presentation. Data are mean ± s.e.m. **h**, As in **g**, for NoGo category-selective neurons. **i**, Scatter plot showing the mean inferred spike rate in correct trials versus incorrect trials, for individual Go (green) and NoGo (red) category-selective neurons. *P*_Go_ = 1.0 × 10^−26^, Pearson’s *r*_Go_ = 0.72, *P*_NoGo_ = 4.1 × 10^−5^, Pearson’s *r*_NoGo_ = 0.52 (*n*_No_ = 156, *n*_NoGo_ = 57). Black line denotes the unity line. Line histogram shows the distribution of difference from unity separately for Go and NoGo-preferring neurons. *P*_Go_ = 6.6 × 10^−22^, *P*_NoGo_ = 1.9 × 10^−5^, two-tailed WMPSR (*n*_Go_ = 156, *n*_NoGo_ = 57). Source data

A possible explanation for the different time courses could be that various task-relevant components differentially contribute to the average selectivity. It is well established that—beyond the category selectivity we observed—the mPFC contains representations of choice and reward^32–35^. In our paradigm, choice and reward associations are learned earlier than categories, and stay constant through the rule-switch. Therefore, neurons selective for choice and reward are expected to show a different time course than neurons selective for stimulus category (Extended Data Fig. 7g). We identified individual neurons that acquire selectivity early-on during task learning as well as neurons that develop selectivity more gradually, with increasing categorization demand (Extended Data Fig. 7h–k). In line with their average (Fig. 3c, d), most NoGo-preferring neurons followed the gradual time course, reflecting acquisition of the respective category rule, whereas Go-preferring neurons followed either of the time courses (Extended Data Fig. 7h–k). Thus, neurons that prefer the Go category were modulated by category, as well as by the earlier learned reward and choice associations (Extended Data Fig. 8).

**Extended Data Fig. 9:**
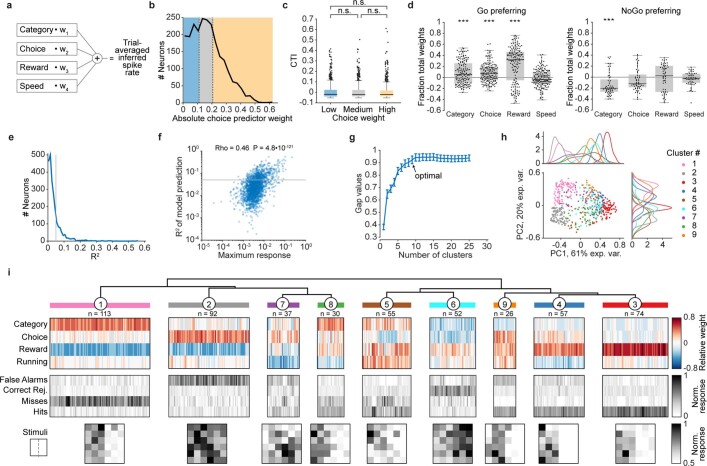
mPFC contains neural correlates of multiple task components. **a**, Linear regression model, fitting the trial-averaged inferred spike rates of individual neurons at T5 (‘*w*_*i*_’ denotes the predictor weight; category predictor 0: category 1; 1: category 2; Methods). **b**, Distribution of absolute choice predictor weight of all observed neurons, divided into low, middle and high weight groups with equal numbers of cells. **c**, Box plots of CTI distributions for the choice weight groups in **b**. Boxes show the first to third quartile of the distributions, and black line denotes the median. There was no significant difference between the distributions, showing that category selectivity is not observed exclusively in highly choice-correlated cells. *P* = 0.92, Kruskal–Wallis test comparing all groups, chi-squared = 0.158, d.f. = 2. **d**, Relative weights of linear regression predictors (category identity, choice, reward and running speed) of Go and NoGo category-selective cells at T5. Left, category, choice and reward predictors show a significant deviation from 0. Right, only the category predictor shows a significant difference from 0. *P*_Go-w1_ = 6.8 × 10^−5^, *P*_Go-w2_ = 2.3 × 10^−7^, *P*_Go-w3_ = 2.0 × 10^−14^, *P*_Go-w4_ = 0.17, *P*_NoGo-w1_ = 3.9 × 10^−5^, *P*_NoGo-w2_ = 0.03, *P*_NoGo-w3_ = 0.68, *P*_NoGo-w4_ = 0.11, two-tailed WMPSR tests, Bonferroni corrected for four comparisons (*n*_Go_ = 156, *n*_NoGo_ = 57 cells). Grey boxes span the first to third quartile, black lines show the median. **e**, Distribution of *R*^2^ values, black line at 0.05 denotes the cut-off for cells included in hierarchical clustering (resulting in 536 out of 2,306 neurons, largely excluding unresponsive neurons). **f**, Correlation of the *R*^2^ value of individual cells and their maximum average response to correct or incorrect trials of either category. *P* = 4.8 × 10^−121^, rho = 0.46, Spearman’s correlation (*n* = 2,306 cells). Grey line denotes the *R*^2^ cut-off shown in **e**, which eliminated mostly unresponsive neurons. **g**, Gap statistic of hierarchical clustering for varying cluster numbers. Arrow denotes the optimal number of clusters (nine clusters; Methods). Error bars denote the standard error of the gap statistic value. **h**, Principal component analysis of model weights shows cluster separation along the major axes of variance. Line histograms show distributions per cluster along PC1 and PC2 separately. Individual neurons (dots) are colour-coded by cluster identity. **i**, Top, dendrogram showing cluster linkage. Second row, for each neuron, relative weights of model predictors in each of the nine clusters. Third row, for each neuron, normalized responses in the four different trial outcomes. Fourth row, per cluster, mean normalized response to every stimulus. Source data

To disentangle how stimulus category, choice and reward affected the trial-by-trial responses of category-selective neurons, we used linear regression to determine their individual contributions (Extended Data Fig. 9a). Although choice selectivity did not directly explain CTI (Extended Data Fig. 9b, c), the activity pattern of Go category-selective cells showed significant modulation by multiple factors, stimulus category, choice and reward (Extended Data Fig. 9d). By contrast, the responses of NoGo category-selective cells were only significantly influenced by category identity (Extended Data Fig. 9d). We performed hierarchical clustering to explore the entire task-responsive neuronal population in the mPFC including category-selective cells and found clusters of mPFC neurons that were predominantly modulated by a single parameter—that is, category, choice (lick) and reward (Extended Data Fig. 9e–i, cluster number 1, 2 and 3, respectively). In addition, there were also clusters of neurons modulated by specific combinations of task parameters (Extended Data Fig. 9i, clusters number 4, 5 and 9). These results are in line with recent studies in primates and mice, reporting mixed selectivity of neurons in the PFC after animals learned cognitive tasks^21,36–38^. In summary, the mouse mPFC contains neurons modulated by a single parameter (such as category) and neurons that show mixed-selective responses.

## Category tuning generalizes across tasks

**Fig. 4:**
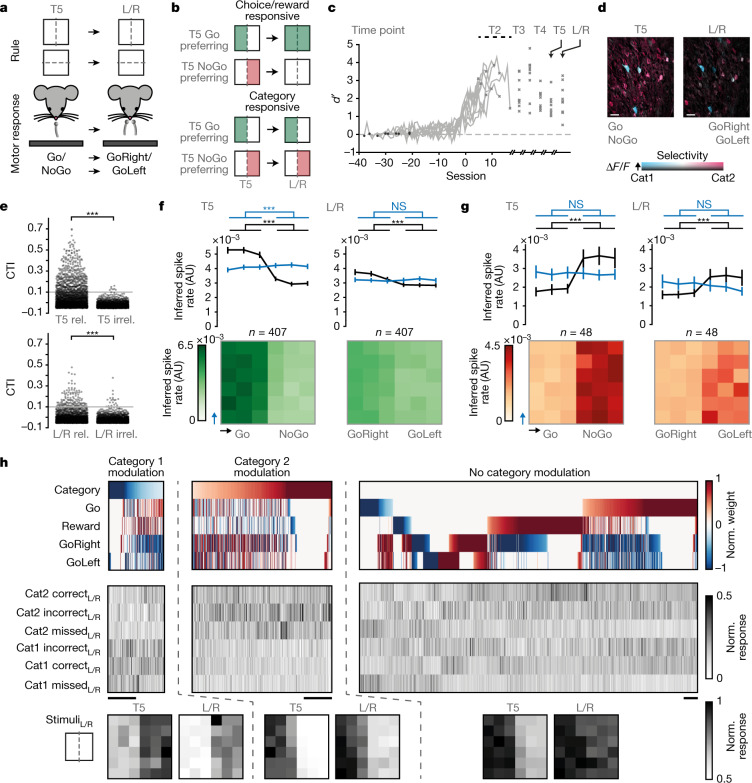
Mouse mPFC contains uniquely category-modulated neurons. **a**, Schematic depicting the change in task from Go/NoGo (T5) to left/right choice (L/R). The motor response changed from Go to GoRight, and from NoGo to GoLeft. The category rule remained the same. **b**, Possible changes in neuronal responses between T5 and left/right choice. Top, choice/reward-selective neurons. Bottom, uniquely category-selective neurons. **c**, *d*′ throughout category learning and the change in task, aligned to criterion (>66% correct, *n* = 9 mice). **d**, Example HLS maps before (T5) and after (L/R) the task change. Scale bars, 30 μm. Hue: preferred category; lightness: response amplitude; saturation: category selectivity. **e**, CTI of all recorded neurons, calculated using either the relevant or the irrelevant rule, before (T5) and after (L/R) the task change. T5: *P* = 1.3 × 10^−161^, L/R: *P* = 1.3 × 10^−24^, two-tailed WMPSR test (*n* = 2,389). Grey lines denote CTI = 0.1. **f**, Top, inferred spike rate for stimuli ordered along the relevant dimension (black), or the irrelevant dimension (blue) across all Go category-selective neurons (defined at T5, CTI > 0.1). *P*_T5 rel_ = 6.5 × 10^−28^, *P*_T5 irrel_ = 1.6 × 10^−5^, *P*_L/R rel_ = 1.6 × 10^−19^, *P*_L/R irrel_ = 0.38, two-tailed WMPSR test (*n* = 407). Data are mean ± s.e.m. Bottom, mean activity per stimulus. **g**, As in **f**, for NoGo category-selective neurons. *P*_T5 rel_ = 1.9 × 10^−4^, *P*_T5 irrel_ = 0.45, *P*_L/R rel_ = 7.7 × 10^−4^, *P*_L/R irrel_ = 0.21, two-tailed WMPSR (*n* = 48). **h**, Predictor weights and response amplitudes of significantly modulated neurons. *P* < 0.05, at least one predictor, *F*-statistic (1,904 neurons). Scale bars, 50 neurons. Top row, normalized predictor weights for each neuron. Left, neurons with a negative category weight (category 1, NoGo/GoLeft). Middle, neurons with a positive category weight (category 2, Go/GoRight). Right, no significant category modulation. Middle row, average (normalized) activity in correct, incorrect and missed trials of categories 1 and 2 separately. Bottom row, per group, the mean normalized response to all presented stimuli at T5 (left) and L/R (right). Source data

Because the activity of many mPFC neurons, including category-selective neurons, correlated with combinations of stimulus category, choice and reward, we aimed to experimentally determine the unique category-selective component. Exclusively category-modulated neurons can be revealed by experimental decoupling of the presented category and the associated motor response^39^. Within the framework of our rule-based categorization paradigm, we achieved this by initially training mice to categorize in the Go/NoGo task (as before), and then changing the task to a left/right choice design (Fig. 4a). As a consequence, the previous Go (lick) category changed into a ‘GoRight’ (lick right) category, and the previous NoGo (no lick) category was now also rewarded if the mouse made a ‘GoLeft’ (lick left) response. In this experiment, neurons that were category-selective in the Go/NoGo task could either retain their category selectivity in the left/right choice task (indicating that they are genuinely category-selective), or change their response pattern, reflecting selectivity rather for motor planning, choice or associated reward (Fig. 4b).

We first trained nine mice to categorize visual stimuli according to either the spatial frequency or the orientation rule (the task was identical to that in Fig. 1 and Extended Data Fig. 1, up to the generalization test T5; Fig. 4c). After session T5, we changed the behavioural setup by replacing the single centred lick spout with two laterally placed lick spouts (left/right choice paradigm). The mice quickly adapted to the change and within the first four trials also responded with licks to the previous NoGo category (now GoLeft; note that the ratio between the left and right licks varied throughout the session). Although the mice did not specifically target their first licks to the correct spout, they performed a similar number of licks on both lick spouts and obtained a similar amount of rewards for both categories.

**Extended Data Fig. 10:**
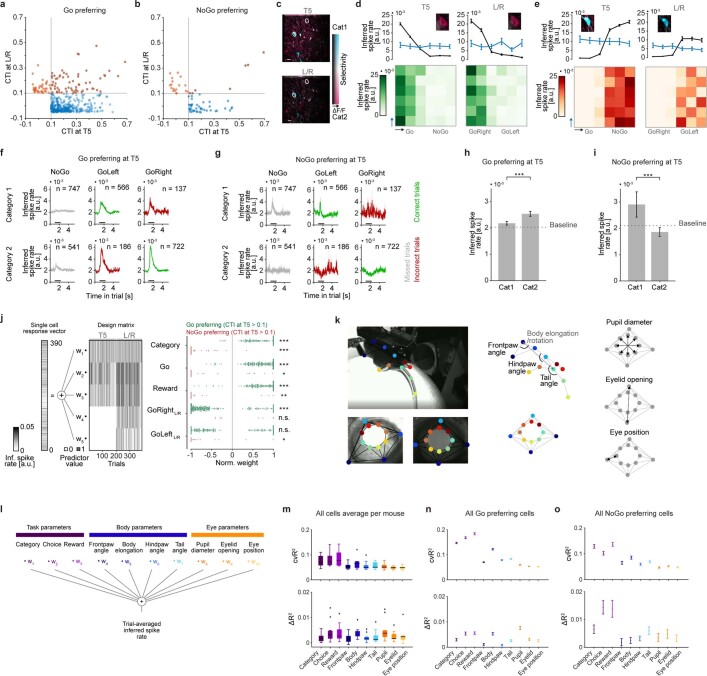
Category selectivity throughout the task change and contributions of task-relevant parameters and uninstructed movements to explained response variance. **a**, Scatter plot showing the CTI of Go-preferring neurons having a CTI > 0.1 in session T5 (blue) or session L/R (orange). Grey lines denote the CTI threshold used to determine category selectivity. **b**, As in **a**, for NoGo preferring cells. **c**, HLS maps of the example imaging region before (T5) and after (L/R) the task change (also shown in Fig. 4). Scale bar, 30 μm. White circles indicate example cells in **d** and **e**. Hue: preferred category; lightness: response amplitude; saturation: category selectivity. **d**, Example Go-preferring neuron. Top, inferred spike rate for stimuli ordered along the relevant dimension (black), or the irrelevant dimension (blue). Inset, section from the HLS map in **c** showing the example cell. Bottom, average inferred spike rate per stimulus. Data are mean ± s.e.m. **e**, As in **d**, but for a NoGo preferring example cell. **f**, Inferred spike rate of Go category-selective neurons (selected at T5), separated by stimulus/trial outcome combination in the left/right choice task. Top row, category 1 (GoLeft is correct). Bottom row, category 2 (GoRight is correct). Grey, missed trials, no reward. Green, rewarded trials. Red, unrewarded trials. Black line, stimulus presentation. In each panel, ‘*n*’ indicates the total number of included trials (from nine mice). Data are mean ± s.e.m. **g**, As in **f**, for NoGo category-selective neurons (determined at T5). **h**, Category-selective neuronal responses, in absence of behavioural responses (missed trials in the left/right choice task). Inferred spike rate for each category presented in the left/right choice task, of Go category-selective neurons selected at T5. *P* = 3.3 × 10^−8^, two-tailed WMPSR (*n* = 407). Data are mean ± s.e.m. **i**, As in **h**, but for NoGo category-selective neurons (determined at T5). *P* = 0.002, two-tailed WMPSR (*n* = 48). **j**, Left and middle, schematic of the linear regression model, fitted to all trials of sessions T5 and 2AC combined. The average trial spike rate of each neuron was predicted by a weighted sum of the predictors: Category, Go, Reward, GoRight and GoLeft. Response vector and design matrix of example session. Right, significant normalized weights of all category-selective cells. *P*_Go-w1_ = 4.6 × 10^−19^, *P*_Go-w2_ = 6.1 × 10^−37^, *P*_Go-w3_ = 5.4 × 10^−43^, *P*_Go-w4_ = 1.1 × 10^−23^, *P*_Go-w5_ = 0.58, *P*_NoGo-w1_ = 2.2 × 10^−6^, *P*_NoGo-w2_ = 0.003, *P*_NoGo-w3_ = 0.002, *P*_NoGo-w4_ = 0.40, *P*_NoGo-w5_ = 0.006, two-tailed WMPSR tests (*n*_Go_ = 407, *n*_NoGo_ = 48). **k**, Left, example cropped image of the body-imaging camera and the eye-imaging cameras, with overlaid marker positions (tracked using DeepLabCut^41,42^). Middle and right, schematics defining body and eye parameters derived from the tracked markers. **l**, Schematic showing predictors and the linear regression model used to fit the cells’ mean inferred spike rates per trial. **m**, Top, box plots showing the maximum predictive power (cv*R*^2^) of each model predictor (*n* = 9 mice). Boxes show the first to third quartile; black line denotes the median. Bottom, box plots showing the unique contribution (∆*R*^2^) of each model predictor. **n**, Maximum predictive power (cv*R*^2^, top) and the unique contribution (∆*R*^2^, bottom) across Go category-selective neurons (*n* = 407 neurons). Data are mean ± s.e.m. **o**, As in **n**, but for NoGo category-selective neurons (*n* = 48 neurons). Source data

We found a significant proportion of category-selective neurons before (T5) and after the task change (left/right; threshold of CTI > 0.1 according to the relevant rule) (Fig. 4d, e). On average, category-selective cells identified at T5 discriminated the stimulus categories also after the task change (Fig. 4d, f, g, Extended Data Fig. 10a–g), although their selectivity decreased. The left/right choice task allowed us to compare trials with different stimulus categories in the absence of choice and reward (missed trials). Neurons that were initially selective for the Go category remained selective for the same stimulus category. Likewise, initial NoGo category-selective neurons, remained only responsive to stimuli of the previous NoGo category (Extended Data Fig. 10h, i).

However, the overall decrease in selectivity after the task change indicated that also choice-and reward-selective neurons were identified as ‘category-selective’ in the Go/NoGo task (Fig. 4b). Because the left/right choice task changed how reward and motor contingencies mapped onto the stimulus space, but did not change the mapping of category identity, we were able to use a regression model to disambiguate these contributions. Only neurons that remained category-selective across the task change will be significantly fitted by the Category predictor. Apparent category-selective neurons—that is, choice- and reward-modulated neurons, will be better predicted by the Go and Reward predictors. This analysis showed that mouse mPFC neurons represent categories in conjunction with reward and choice. Most importantly, it also revealed a set of uniquely category-modulated neurons in the mPFC (4.3%) (Fig. 4h, Extended Data Fig. 10j).

Recent work has shown the influence of uninstructed behaviours, such as whisking and eye movements, on neuronal response variability in operant tasks^40^. If such behaviours correlated with the category identity of the presented stimuli, they could lead to apparent category selectivity. To control for this, we tracked key postural markers using DeepLabCut^41,42^ and combined them with in-task recorded instructed behaviours and task parameters to predict neural activity. We found that there was a significant and unique contribution for all instructed and uninstructed behavioural variables. Notably, however, there was also a unique contribution of the category component that could not be accounted for by any of the instructed or uninstructed behavioural parameters (Extended Data Fig. 10k–o, Supplementary Video 1). We therefore conclude that the mPFC contains a sparse but distinct set of neurons that represent learned categories irrespective of associated motor behaviours and reward.

## Discussion

Using a paradigm to study learning of rule-based categories in mice, we could follow neuronal populations in the mPFC throughout the entire learning process, from naive to expert mice. We found two distinct groups of cells developing a representation of learned categories with different learning-related dynamics. The NoGo category representation emerged gradually, was rule-specific and was not strongly modulated by additional task parameters, in contrast to the Go representation. In addition, we observed that selectivity for the Go category increased further in the fast rule-switch phase compared to the slow, initial learning phase. This difference could be a consequence of Go category-selective neurons belonging to intrinsically different representations of choice, reward and categories. By experimentally decoupling these, we confirmed that many category-selective neurons were actually mixed-selective, which could benefit the representation of task-relevant information^38^. However, the experiment also revealed uniquely category-selective mPFC neurons, for both learned categories. In line with previous studies^3,19,32,43^, we found that the mPFC initially contains a conjunctive stimulus and choice representation. This representation flexibly followed the novel Go category when a mouse learned the second rule. In parallel, a slowly learning group of Go category-selective cells emerged for each rule, following a time course similar to the NoGo category representation.

This mouse model of rule-based category learning opens up possibilities to causally investigate neuronal interactions across several cortical and subcortical circuits. Many brain areas, such as posterior parietal cortex^4,44^, sensory areas (P.M.G., S.R., T.B. and M.H., manuscript submitted)^44,45^ and striatum^3^, contribute to multiple aspects of category learning and categorization behaviour. Several circuit models on areal interactions have been put forward^43,46^. One model of particular interest proposed that slow-learning PFC circuits acquire category selectivity using rapidly learned stimulus-specific activity originating in the striatum as a teaching signal^3,46^. Within this framework, the mPFC could compute the rule-dependent NoGo category representation from the fast-arising activity of conjunctive Go/choice-selective neurons mediated by local inhibitory circuits. Rule-based category learning in mice allows for testing of specific predictions of such circuit models for prefrontal cortex function by observing initially naive mice throughout the learning process. In particular, the possibility to investigate and observe neuronal responses in the mPFC during category learning in mice opens a window to study the neural circuitry that underlies categorization and storage of semantic memories^47^ also in this species.

## Methods

### Data reporting

No statistical methods were used to predetermine sample size. Mice were randomly assigned to the categorization rule ‘spatial frequency’ or ‘orientation’. The investigators were not blinded to allocation during experiments and outcome assessment.

### Animals

All procedures were performed in accordance with the institutional guidelines of the Max Planck Society and the local government (Regierung von Oberbayern). Twenty female C57BL/6 mice (postnatal day (P) 63–P82 at the day of surgery) were housed in groups of four to six littermates in standard individually ventilated cages (IVC, Tecniplast GR900). Mice had access to a running wheel and other enrichment material such as a tunnel and a house. All mice were kept on an inverted 12 h light/12 h dark cycle with lights on at 22:00. Before and during the experiment, the mice had ad libitum access to standard chow (1310, Altromin Spezialfutter). Before the start of behavioural experiments, mice had ad libitum access to water. At the end of the experiments, mice were perfused with 4% paraformaldehyde (PFA) in PBS and their brains were post-fixed in 4% PFA in PBS at 4 °C.

### Surgical procedures

Before surgery, a prism implant was prepared by attaching a 1.5 mm × 1.5 mm prism (aluminium coating on the long side, MPCH-1.5, IMM photonics) to a 0.13 mm thick, 3 mm diameter glass coverslip (41001103, Glaswarenfabrik Karl Hecht) using UV-curing optical glue (Norland optical adhesive 71, Norland Products) and was left to fully cure at room temperature for a minimum of 24 h. Mice were anaesthetized with a mixture of fentanyl, midazolam and medetomidine in saline (0.05 mg kg^−1^, 5 mg kg^−1^ and 0.5 mg kg^−1^ respectively, injected intraperitoneally). As soon as sufficient depth of anaesthesia was confirmed by absence of the pedal reflex, carprofen in saline (5 mg kg^−1^, injected subcutaneously) was administered for general analgesia. The eyes were covered with ophthalmic ointment (IsoptoMax/Bepanthen) and lidocaine (Aspen Pharma) was applied on and underneath the scalp for topical analgesia. The scull was exposed, dried and subsequently scraped with a scalpel to improve adherence of the head plate. The scalp surrounding the exposed area was adhered to the skull using Histoacryl (B. Braun Surgical). A custom-designed head plate was centred at ML 0 mm, approximately 3 mm posterior to bregma, attached with cyanoacrylate glue (Ultra Gel Matic, Pattex) and secured with dental acrylic (Paladur). A 3 mm diameter craniotomy, centred at anterior–posterior (AP) 1.9 mm, medial–lateral (ML) 0 mm, was performed using a dental drill. The hemisphere for prism insertion was selected based on the pattern of bridging veins. Before inserting the prism, two injections (50 nl min^−1^) of 200–250 nl of virus solution (AAV2/1.hSyn.mRuby2.GSG.P2A.GCaMP6m.WPRE.SV40, titre: 1.02 × 10^13^ genome copies (GC) ml^−1^, Plasmid catalogue 51473, Addgene) were targeted at the medial prefrontal cortex opposite to the prism implant, coordinates: AP 1.4 mm to AP 2.8 mm, ML 0.25 mm, dorsal–ventral (DV) 2.3 mm (Nanoject, Neurostar). The left hemisphere was injected in 11 mice, and the right hemisphere in 9 mice. Subsequently, a durotomy was performed using microscissors (15070-08, Fine Science Tools) over the contralateral hemisphere, next to the medial sinus. The prism implant was inserted, gently pushing the medial sinus aside until the target cortical region became visible through the prism (for a detailed description, see ref. ^29^). The coverslip was attached to the surrounding skull using cyanoacrylate glue and dental acrylic. After surgery, the anaesthesia was antagonized with a mixture of naloxone, flumazenil and atipamezole in saline (1.2 mg kg, 0.5 mg kg^−1^ and 2.5 mg kg^−1^ respectively, injected subcutaneously) and the mice were placed under a heat lamp for recovery. Post-operative analgesia was provided for two subsequent days with carprofen (5 mg kg^−1^, injected subcutaneously).

### Visual stimuli

Stimuli for behavioural training were presented in the centre of a gamma corrected LCD monitor (Dell P2414H; resolution: 1,920 by 1,080 pixels; width: 52.8 cm; height: 29.6 cm; maximum luminance: 182.3 Cd m^−2^). The centre of the monitor was positioned at about 0° azimuth and 0° elevation at a distance of 18 cm, facing the mouse straight on. The stimuli were 36 different sinusoidal gratings, each with a specific orientation and spatial frequency combination, shown in full contrast on a grey background (see Extended Data Fig. 1 for schematic of stimuli and task stages). Orientations ranged from 0° to 90°, the spatial frequencies from 0.023 cycles per degrees (cyc/°) to 0.25 cyc/° (orientations: [0, 15, 30, 60, 75, 90] °, spatial frequencies: [0.023, 0.027, 0.033, 0.06, 0.1, 0.25] cyc/°). The stimulus size was 45 retinal degrees in diameter, including an annulus of 4 degrees blending into the equiluminant grey background. The gratings drifted with a temporal frequency of 1.5 cycles per s.

In a subset of experiments (*n* = 3 mice), a dense stimulus space was presented, consisting of 49 stimuli ranging from 15° to 75° in orientation and from 0.027 cyc/° to 0.1 cyc/° in spatial frequency (orientations: [15, 30, 37.5, 45, 52.5, 60, 75]°, spatial frequencies: [0.027, 0.033, 0.036, 0.043, 0.052, 0.06, 0.1] cyc/°). Stimuli on the category boundary (either having an orientation of 45° or a spatial frequency of 0.043 cyc/°) were assigned to both categories, hence rewarded in 50% of trials.

All stimuli were created and presented using the Psychophysics Toolbox extensions of MATLAB^48–50^.

### Behaviour

Behavioural experiments started seven days after surgery. The water restriction regime and the behavioural apparatus were previously described^51^. In short, mice were restricted to 85% of their initial weight on the starting date by individually adjusting the daily water ration. First, mice were accustomed to the experimenter and head fixation in the setup by daily handling sessions lasting 10 min. During these sessions, the water ration was offered in a handheld syringe. The remainder was supplemented in an individual drinking cage after a delay of approximately 30 min. After four to seven days of handling, mice were pre-trained to lick for reward, while being head-fixed on the spherical treadmill^52–54^ in absence of visual stimulation. Whenever a mouse ceased to run (velocity below 1 cm s^−1^) and made a lick on the spout, a water reward (drop size 8 μl) was delivered via the spout. A baseline imaging time point (T1) was acquired once the mice consumed more than 50 drops per session (35 to 45 min) on two consecutive days (requiring about three days of pre-training).

Subsequently, daily sessions of visual discrimination training for two initial stimuli started. Each mouse was randomly assigned to one of two groups. One group was first trained on the orientation rule, then on the spatial frequency rule. For the other group, the sequence of the rules was reversed (Extended Data Fig. 1). Each rule defined a Go category and a NoGo category, separated by a boundary at either 45° (orientation rule) or at 0.043 cyc/° (spatial frequency rule). Trials started with an inter-trial interval of 5 s. After that, the mouse could initiate stimulus presentation by halting and refraining from licking for a minimum of 0.5 s. A single stimulus was subsequently shown for 1.3 ± 0.2 s. At any time during stimulus presentation, the mouse could make a lick to indicate a Go choice. Trials with a Go choice in response to a Go category stimulus triggered a water reward and were classified as hits; trials in which the mice failed to lick during Go category stimulus presentation were considered misses. Correct withholding of a lick to a NoGo category stimulus was classified as a correct rejection, and did not result in a water reward. A lick during a NoGo category stimulus counted as a false alarm. Initially, false alarms only led to the termination of the current trial; later during training, false alarms were followed by a time-out of 5–7 s showing a time-out stimulus (a narrow, horizontal, black bar). Time-outs were included to reduce a Go bias that mice typically showed. The second imaging session (T2) was carried out after a mouse performed at more than 66% correct Go choices in a given session (requiring 11 to 40 sessions).

For the next training stage (leading up to imaging session T3) further stimuli were added (Extended Data Fig. 1a), such that both the Go category and the NoGo category consisted of three stimuli differing in the feature either irrelevant to the category rule (T3a, *n* = 6 mice), or relevant to the category rule (T3b, *n* = 5 mice). Whenever a mouse’s performance exceeded 66% correct Go choices in one session, we proceeded to the next training (and imaging) stage; 6 stimuli per category, 9 stimuli per category (imaging session T4), and finally 18 stimuli per category (imaging session T5), the latter serving as a crucial test for generalization behaviour.

Rule-switch: After successful learning of rule 1, mice (*n* = 11) were retrained using the previously irrelevant dimension. This stage, known as rule-switch training, started with two exemplar stimuli for the new rule, and then proceeded with the same steps as for rule 1 and ended with another generalization test of rule 2 (18 stimuli per category, imaging session T8).

Task change: After successful learning of rule 1 (T5), the categorization performance of mice (*n* = 9) was tested with a different operant response, in a left/right choice task. For this session, the behavioural setup was slightly modified to create a left/right choice task. Instead of one lick spout centred in front of the mouse, the mouse was now presented with two lick-spouts, one offset to the left and one offset to the right. Stimuli of the previous Go category were assigned a new GoRight response (rewarded after a lick on the right lick spout). Stimuli of the previous NoGo category were assigned a new GoLeft response (rewarded after a lick on the left lick spout). The original stimulus to category assignment—that is, the categorization rule—remained the same throughout the task change. Before the first stimulus presentation, ten drops were manually given on each lick spout to motivate the mice to lick on both sides.

Throughout training, stimuli from the Go category and the NoGo category were presented in a pseudorandomized fashion, showing not more than three stimuli of the same category in a row. The behavioural training program was a custom written MATLAB routine (Mathworks).

### Imaging

Two-photon imaging^55^ through the implanted prism was performed at 5–8 time points in each mouse throughout the training paradigm (T3 was omitted in two mice; for detailed timing of the imaging sessions see Extended Data Fig. 1a). In some mice (*n* = 5) we followed two regions in the same mouse; in these cases, two imaging sessions were acquired on consecutive days during the same training stage. Imaging was done using a custom-built two-photon laser-scanning microscope (resonant scanning system) and a Mai Tai eHP Ti:Sapphire laser (Spectra-Physics) tuned to a wavelength of 940 nm. Images were acquired with a sampling frequency of 10 Hz and 750 × 800 pixels per frame. The mice in the task change experiment were imaged using a customized commercially available two-photon laser-scanning microscope (Thorlabs; same laser specifications as described above), operated with Scanimage 4^56^. In these experiments, images were acquired at 30 Hz and 512 × 512 pixels per frame. The average laser power under the objective ranged from 50 to 80 mW. Note that the laser power was higher than for imaging through a conventional cranial window due to a substantial power loss over the prism^29^. We used a 16×, 0.8 NA, water immersion objective (Nikon) and diluted ultrasound gel (Dahlhausen) on top of the implant as immersion medium. Two photomultiplier tubes detected the red fluorescence signal of the structural protein mRuby2 (570–690 nm) and the green fluorescence signal of GCaMP6m (500–550 nm)^57^. During imaging, the monitor used for stimulus presentation was shuttered to minimize light contamination^58^. The imaging data were acquired using custom LABVIEW software (National Instruments; software modified from the colibri package by C. Seebacher) and the synchronization of imaging data with behavioural readout and stimulus presentation was done using DAQ cards (National Instruments).

### Tracking of postural markers

In two-photon imaging sessions of a subset of experiments, the mouse was video-tracked using infrared cameras (The Imaging Source Europe). Two cameras were aimed at the eyes, and a third camera was positioned at a slight angle behind the mouse, in order to record body movements in-task. The eyes of the mouse were back-lit by the infrared two-photon imaging laser and the body was illuminated using an infrared light source (740 nm; Thorlabs). Key eye and body features (see Extended Data Fig. 10) were manually defined and automatically annotated using DeepLabCut^41,42^. From the *x* and *y* coordinates of these features, we calculated three eye parameters and four postural parameters (pupil diameter, eye position, eyelid opening, front paw angle, hind paw angle of the left hind paw, body elongation/rotation, tail angle; see Extended Data Fig. 10). Supplementary Video 1 shows both eye and body cameras of an example mouse.

### Data analysis

The analysis of behaviour and imaging data was performed using custom written MATLAB routines.

### Behavioural data

Behavioural performance is shown as the sensitivity index, *d*′. For every training session, *d*′ was calculated as the difference between the *z*-scored hit rate and the *z*-scored false alarm rate. The hit rate was defined as the number of correct category 2 trials divided by the total number of category 2 trials per session. Similarly, the false alarm rate was calculated as the number of incorrect category 1 trials divided by the total number of category 1 trials. In case a mouse performed two training sessions at time points T1, T3, T4, T5, T7 and T8, because two regions were imaged, the displayed value in the learning curve is the average across the two imaging sessions.

The fraction of correct Go choices was calculated as the number of hit trials divided by the number of all trials in which the mouse made a Go choice (the sum of ‘hits’ and ‘false alarms’). The number of days until a mouse reached performance criterion was the amount of daily sessions until the fraction of correct Go choices exceeded 0.66. Pre-training sessions without visual stimulation were not included in this quantification.

To investigate categorization behaviour across the entire stimulus space, we calculated the ‘fraction chosen’: The number of Go choices in response to a specific stimulus divided by the total number of presentations for that stimulus (see example in Fig. 1d; for all mice see Extended Data Fig. 2). Finally, we constructed psychometric curves showing the effect of each feature (that is, rule-relevant versus rule-irrelevant) on the behaviour of the mice (Fig. 1j). For that, the stimulus-specific ‘fraction chosen’ values were averaged along the irrelevant or the relevant feature dimension, respectively (see Fig. 1i).

To estimate learning rates, each individual learning curve was fitted with a sigmoid function:

$$y(x)=p1+\frac{p2}{1+{{\rm{e}}}^{p3(x-p4)}}$$

in which *p*1 determines the minimum of the sigmoid curve (for curve fitting fixed to 0), *p*2 the maximum, *p*3 the slope and *p*4 the inflection point. The parameter defining the minimum was fixed at a *d*′ of 0. Learning curves for rule 1 and rule 2 were fitted independently. Goodness of fit was determined as the root-mean-square error between the learning curve and the fitted curve.

### Imaging data processing

The imaging data were first preprocessed by performing dark-current subtraction (using the average signal intensity during a laser-off period) and line shift correction. Rigid *xy* image displacement was first calculated on the structural red fluorescence channel using the cross correlation of the 2D Fourier transform of the images^59^, and subsequently corrected on both channels. For each imaging session, cells were manually segmented using the average image of the red fluorescence channel across the entire session. The cell identity was then manually matched across all imaging time points and only cells that could be identified in every session from T1 to T8 or T5 to left/right were included in the analysis. This criterion excluded one mouse (M06) from all further analyses, because of lost optical access at T8. The average green fluorescence signal was extracted for each cell and then corrected for neuropil contamination by subtracting the signal of 30 μm surrounding each cell multiplied by 0.7 and adding the median multiplied by 0.7 (refs. ^57,60^). From this fluorescence trace, we calculated Δ*F*/*F* as (*F* − *F*_0_)/*F*_0_ per frame. *F*_0_ was defined as the 25th percentile of the fluorescence trace in a sliding window of 60 s. From this trace, we inferred the spiking activity of each cell using the constrained foopsi algorithm^61–63^. The inferred spike rate during the stimulus presentation period was used for all further calculations and in all figure panels, except for the HLS maps and the left panels of Fig. 2d, e, where we display the Δ*F*/*F* trace for comparison.

To display lick-triggered neuronal activity (Extended Data Fig. 8), we averaged the inferred spike rate centred on the onset of the mouse’s lick-bouts. A lick-bout was defined as a sequence of licks, in which the interval between every two consecutive licks did not exceed 500 ms. Thus, a lick was part of a lick-bout if it happened within 500 ms after the previous lick. The onset of each lick-bout was the time of the first lick in the lick-bout.

### Category-tuning index

For every cell, we calculated the CTI as previously described^30^. In short, we quantified the mean inferred spike rate during stimulus presentation for every stimulus. Next, we calculated the mean difference in inferred rate between stimuli of the same category (within), subtracted it from the mean difference between stimuli belonging to the two different categories (across) and normalized by the sum (across + within). This calculation results in an index ranging from −1 to 1, with category-unselective cells showing CTIs close to and below 0 and an ideal category-selective cell having an index of 1. Category-selective cells were defined as cells with a CTI value larger than 0.1. This threshold was chosen based on the distribution of CTIs in the naive population (T1), where individual cells rarely crossed this value. As a control, we used other thresholds (0.07, 0.15 and 0.20) and found no qualitative difference in the results other than that the fraction of category-selective cells scaled.

The fraction of category-selective cells was calculated as the number of neurons above threshold per imaging region, divided by the total number of chronically recorded neurons in that imaging region. Category-selective cells, determined by their CTI at time points T5 and T8, were divided in a Go category-selective and a NoGo category-selective group; neurons with higher average activity in Go category trials than in NoGo category trials were grouped as Go category-selective cells and conversely, cells with a higher average activity in NoGo category trials were labelled as NoGo category-selective. The overlap between the Go and NoGo category-selective groups was calculated between T5 and T8. The expected range of overlap assuming random independent sampling was calculated from the data, but with shuffled neuron identities (using the 95% percentile of the shuffled distribution). For time points at which not all stimuli were presented (T2, T3, T4, T6 and T7), we approximated category-tuning from the average responses to Go category trials and NoGo category trials.

### Bayesian decoding

We decoded category identity from trial-by-trial activity patterns of a single neuron up to groups of ten neurons using Bayes theorem:

$$p(c|r)=\frac{p(r|c)p(c)}{p(r)}$$

in which *p*(*r*|*c*) is the probability of a single trial response *r* when observed in either category 1 or 2 trials (calculated from an exponential distribution), *p*(*c*) as the prior probability of observing each category, and *p*(*r*) as the probability of observing the response. To cross-validate decoding performance, trials were first split into a training and test set (70% and 30%, respectively). The trial-averaged inferred spike rates followed an exponential distribution, which we estimated for each category individually (using the training set). Then, for each trial in the test set, we calculated the probability that the neuronal response came from those distributions. The distribution that gave the higher probability was determined as the decoder’s prediction. Decoder performance was calculated as the fraction of correctly predicted trials. As a control, decoding performance was also calculated after shuffling category identities across trials.

### Selectivity time course

Average selectivity of individual neurons was calculated as the mean difference between responses to all Go category stimuli and all NoGo category stimuli, at every imaging time point (T1–T8). For linear regression, we defined three characteristic selectivity time courses (shown in Extended Data Fig. 7), resembling acquired selectivity for reward/choice, categorization rule 1 and categorization rule 2. Within each of these time courses, maximum selectivity was assigned the value 1 and no selectivity the value 0. The characteristic time courses were used as predictors in a model fitting the development of selectivity of individual neurons over time.

### Generalized linear models to assess the influence of individual task parameters

We performed multilinear regression on neurons that were identified in all imaging time points of the rule-switch experiment. The regression model predicted the trial-wise mean spike rate of each cell during the stimulus presentation periods at imaging time point T5. Categorical predictors were: Category identity of the presented stimulus (0: category 1, 1: category 2), choice of the mouse (0: NoGo, 1: Go), and reward (0: no reward, 1: reward). The average running speed during the trial was modelled as a continuous predictor. A positive predictor weight indicated that the activity of a neuron was increased in trials where the value of the predictor was higher. A negative predictor weight reflected an inverse relation between the predictor’s value and the neuron’s firing rate. We normalized the predictor weights for overall differences in response amplitudes, by dividing each weight by the sum of all absolute predictor weights (including the intercept).

Hierarchical clustering was performed on relative predictor weights of neurons, including only cells with an *R*^2^ value larger than 0.05. The optimal number of clusters was calculated using gap statistic values, determined as the smallest cluster number k that fulfilled the criterion (here nine clusters):

$${\rm{Gap}}(k)\ge {\rm{Gapmax}}-{\rm{s.}}{\rm{e.}}\,({\rm{Gapmax}})$$

in which Gap(*k*) is the gap statistic for *k* clusters, Gapmax is the largest gap value, and s.e.(Gapmax) is the standard error corresponding to the largest gap value.

We obtained linkage and relative predictor weights of the clusters from the MATLAB clusterdata algorithm.

To probe the influence of operant motor behaviour in the task change experiment, we concatenated all trials of sessions T5 (generalization session, Go/NoGo task) and L/R (left/right choice task). A stepwise linear regression model predicted the trial-averaged inferred spike rate of all recorded neurons individually. The predictors were the following categorical variables: category identity of the stimulus (0: category 1; 1: category 2), Go response of the mouse (0: NoGo, 1: all forms of Go, that is, Go/GoRight/GoLeft), reward (0: no reward, 1: reward) and two predictors that were specific to a motor response in the left/right session: GoRight and GoLeft. We only considered significant predictor weights, determined from an *F*-statistic comparing a model with and without a predictor. Predictor weights were normalized by dividing each weight by the maximum of all predictor weights.

### Linear regression assessing the influence of instructed and uninstructed behaviours

The trial-averaged inferred spike rate of all recorded neurons in session T5 of a subset of experiments was fitted using a linear model. Body and eye parameters describing uninstructed behaviours were included in the model as continuous predictors. In addition, we included three categorical task-relevant predictors: category identity of the presented stimulus, choice of the mouse, and reward. For each predictor, we determined its maximum predictive power (cv*R*^2^) and its unique contribution (Δ*R*^2^), similar to the approach previously described^40^. Maximum predictive power (cv*R*^2^) was calculated as the predictive performance (*R*^2^) of a model with all parameters shuffled, except for the parameter of interest. A parameter’s unique contribution (Δ*R*^2^) was quantified as the difference between the full model’s *R*^2^ and the *R*^2^ of a model in which the parameter of interest was shuffled.

### Stereotaxic coordinates of imaging regions

We determined the stereotaxic coordinates of the centres of all imaging regions (included in Fig. 2g, h) to place the imaged regions within a common reference frame (Mouse Brain Atlas)^64^. First, we cut 60-μm thick sagittal sections of both hemispheres using a freezing microtome. The AP coordinates outlining the full extent of the prism were identified from a section of the hemisphere into which the prism had been implanted (Extended Data Fig. 4). On the basis of this information, we calculated the exact AP coordinate of the centre of each imaging field of view. We calculated the dorso-ventral coordinate relative to the brain surface, which was aligned with the dorsal border of the prism. Finally, we determined the medio-lateral coordinate of the imaged field of view from the imaging depth of the field of view relative to the medial pia mater.

### Statistical procedures

All data are presented as mean ± s.e.m. unless stated otherwise. Tests for normal distribution were carried out using the Kolmogorov–Smirnov test. Normally distributed data were tested using the two-tailed paired-samples *t*-test. Non-normally distributed data were tested using the two-tailed WMPSR test for paired samples, and the Kruskal–Wallis test for multiple, independent groups. A Bonferroni alpha correction was applied when multiple tests were done on the same data. Correlations were assessed using Pearson’s correlation coefficient, if the data were normally distributed along both axes; otherwise, Spearman’s correlation was applied.

### Reporting summary

Further information on research design is available in the Nature Research Reporting Summary linked to this paper.

## Online content

Any methods, additional references, Nature Research reporting summaries, source data, extended data, supplementary information, acknowledgements, peer review information; details of author contributions and competing interests; and statements of data and code availability are available at 10.1038/s41586-021-03452-z.

## Supplementary information

Reporting Summary

Video 1 Tracking of eye- and body features in the categorization task Recordings of both eyes and body of an example animal performing in session T5, shown is an excerpt of four trials. Top: Animal body, overlaid with DeepLabCut^41,42^ annotated features. The red or green square appears during presentation of category 1 or 2, respectively. The blue square appears when the animal makes licks. Bottom left: Right eye, annotated with key features. Bottom right: Left eye, annotated with key features.

## Data Availability

The data supporting the findings of this study are available on publication at https://gin.g-node.org/sreinert/Category-learning_mPFC. Source data are provided with this paper.

## Source data

Source Data Fig. 1 Source Data Fig. 2 Source Data Fig. 3 Source Data Fig. 4 Source Data Extended Data Fig. 1 Source Data Extended Data Fig. 2 Source Data Extended Data Fig. 3 Source Data Extended Data Fig. 4 Source Data Extended Data Fig. 5 Source Data Extended Data Fig. 6 Source Data Extended Data Fig. 7 Source Data Extended Data Fig. 8 Source Data Extended Data Fig. 9 Source Data Extended Data Fig. 10

## Code availability

The custom written MATLAB routines used for data collection and analysis are available upon reasonable request.
